# Dysfunctional breathing in patients with moderate and severe obstructive sleep apnea: a cross sectional study

**DOI:** 10.1007/s11325-026-03673-4

**Published:** 2026-05-01

**Authors:** Mrudula Pawar, Prem Venkatesan, Satyanarayana Mysore, Guruprasad Bhat, Vani Lakshmi R.

**Affiliations:** 1 Department of Physiotherapy, Manipal College of Health Professions, Manipal Academy of Higher Education, Bangalore, Manipal, Karnataka India; 2 Department of Pulmonology, Manipal Hospital, Bangalore, Karnataka India; 3 Department of Data Science, Prasanna School of Public Health, Manipal Academy of Higher Education, Manipal, Karnataka India

**Keywords:** Obstructive sleep apnea, Dysfunctional breathing, Breathing pattern, Mouth breathing

## Abstract

**Purpose:**

The nocturnal breathing alterations among patients with obstructive sleep apnea (OSA) impact their daytime breathing mechanics. However, dysfunctional breathing remains under-recognized with limited evidence in these patients. The study aimed to examine the relationship between dysfunctional breathing (DB) and apnea hypopnea index (AHI) among patients with moderate and severe OSA. The secondary objective was to compare DB between patients with moderate and severe OSA.

**Methods:**

A cross sectional study was performed at Manipal Hospital, Bangalore with 120 participants. The biomechanical component, biochemical component and patient reported symptoms of DB were assessed using manual assessment of respiratory motion (MARM), breath holding test (BHT) and self evaluation of breathing questionnaire (SEBQ), respectively. Correlation and regression analysis were used to determine the relationship between DB and AHI. Comparison of outcomes among individuals with moderate OSA and severe OSA was done.

**Results:**

MARM volume showed non-significant negative correlation with AHI with ρ = -0.085. MARM Balance and ribcage motion showed significant correlation (*p* < 0.05) with ρ = 0.187 and ρ = 0.205, respectively. The BHT showed non-significant correlation (*p* > 0.05) with ρ = -0.105 and patient reported symptoms showed significantly positive correlation with ρ = 0.458. The regression model showed significant findings on adjusting for the potential confounders. Also, significantly deteriorated dysfunctional breathing outcomes were reported in patients with severe OSA.

**Conclusion:**

The DB outcomes showed marked relationship with the severity of OSA. Additionally, all the aspects of DB were substantially affected in individuals with severe OSA as compared to those with moderate OSA.

**Supplementary Information:**

The online version contains supplementary material available at 10.1007/s11325-026-03673-4.

## Introduction

Obstructive sleep apnea (OSA) is a major public health concern characterized by repeated episodes of apnea and hypopnea during sleep, resulting in intermittent hypoxia and sleep fragmentation [1]. The disturbed sleep further leads to excessive daytime sleepiness, increased risk of accidents at workplace, road traffic accidents, and other cardiovascular, metabolic and neurocognitive comorbidities [2]. OSA contributes to a considerable socioeconomic burden worldwide with approximately 936 million adults between 30 and 69 years of age suffering from OSA [3]. Similarly, a recent cross sectional study reported that moderate to severe OSA is prevalent in about one fourth of women and one third of men in India and the prevalence increases significantly after 50 years of age [4].

The apnea and hypopnea episodes in OSA could be attributed to the collapsible upper airways during sleep. The anatomical cause behind these involve narrowing of the oropharyngeal space, deposition of fat around pharynx, craniofacial abnormalities and increased tongue fat. In addition to this, the functional mechanisms such as reduced responsiveness of airway dilator muscles, low arousal threshold and ventilatory instability contribute to the pathophysiology of OSA [5]. The disruption in the normal airflow due to upper airway obstruction prompts the compensatory modifications in the respiratory timing to maintain ventilation [6]. Jack et al. [7] suggested that these maladaptive ventilatory responses may eventually become ingrained within an individual’s respiratory control system. Such abnormalities may exacerbate ventilatory instability and perpetuate the cycle of airway collapse, arousal, and sympathetic activation [8]. Moreover, chronic disturbances in breathing pattern could lead to daytime symptoms such as fatigue, dyspnea, and decreased functional performance [9].

A study by Bloch et al., reported that inspiratory airflow limitation during sleep especially in snorers is associated with asynchrony in ribcage and abdominal motion with a predominant thoracic breathing [10]. Similarly, research has demonstrated the variability in the respiratory patterns for patients with OSA and mixed sleep apnea during wakefulness by recording and analyzing the respiratory signals from polysomnography before sleep onset [11]. While informative, this approach may not capture the genuine awake breathing pattern, as measurements taken within the polysomnography setting are influenced by pre-sleep physiological adjustments, postural effects, and the presence of monitoring equipment. In line with this, a recent study assessed the breathing instability during daytime in patients with OSA using breathing rate variability outcome [12], however, the alterations in breathing pattern was not assessed.

According to Vidotto et al., [9] the alterations in breathing pattern are characteristic of dysfunctional breathing. Dysfunctional breathing is a multidimensional term with a breathing pattern component, biochemical component and patient reported breathing symptoms and these three components do not necessarily co-exist in the same individual [13]. The breathing pattern parameter is evaluated through the thoracic and abdominal movements during normal breathing, as the predominant thoracic breathing or the asynchrony suggests disturbed pattern [13]. The biochemical component determines the ventilatory control by assessing the end tidal CO_2_ or duration of end expiratory breath hold [13]. Additionally, the self reported questionnaires assess the respiratory specific symptoms experienced by the patients [13].

Despite the available findings, dysfunctional breathing remains under-recognized among patients with OSA. There is a dearth in literature that determines breathing dysfunction in OSA or examines its association with disease severity. Addressing these relationships could enhance the understanding of the pathophysiology of OSA and may have important implications for rehabilitation strategies, including breathing retraining targeting both mechanical and functional components of respiration. Therefore, we hypothesize that the components of dysfunctional breathing could be associated with the severity of OSA. The current study aimed to examine the relationship between dysfunctional breathing and apnea hypopnea index (AHI) among patients with moderate and severe OSA. The secondary objective of the study was to compare the components of dysfunctional breathing between patients with moderate and severe OSA.

## Methodology

### Study design and study setting

A cross sectional study was performed at Manipal Hospitals, Bangalore between March, 2025 and August, 2025. The study was approved by the Ethical committee of Manipal Hospital, Bangalore and was conducted according to the Declaration of Helsinki. The study was registered at Clinical Trials Registry – India (CTRI/2025/03/082574). Informed consent was obtained from all the participants prior to their participation in the study. The study is reported in accordance with “The Strengthening the Reporting of Observational Studies in Epidemiology (STROBE) Statement: guidelines for reporting observational studies”.

## Participants

Male and female patients with 25 to 65 years of age, body mass index < 40 kg/m^2^ and diagnosed with moderate to severe OSA (AHI ≥ 15 events/hour) according to the American academy of sleep medicine (AASM) guidelines [14] were included in the study. The exclusion criteria were patients with any obstructive nasal conditions such as allergic rhinitis, any comorbid sleep disorders, any craniofacial malformations, history of pharyngeal surgery, stroke, chronic obstructive pulmonary disease, congestive heart failure, or neuromuscular disorders.

## Outcomes

The outcomes assessed were apnea hypopnea index (AHI), Manual assessment of respiratory motion (MARM), breath holding test (BHT) and self evaluation of breathing questionnaire (SEBQ).

The participants underwent a home sleep apnea testing (HSAT) overnight to assess the AHI and ODI. It was in accordance with the AASM recommendations [15]. The device followed the SCOPER (Sleep, Cardiovascular, Oximetry, Position, Effort and Respiratory) criteria with S_0_, C_3_, O_1X_, P_2_, E_1_, R_2_ [16].

According to Rosalba Courtney, dysfunctional breathing is a multidimensional term with a breathing pattern component, biochemical component and patient reported breathing symptoms [13]. So MARM, BHT and SEBQ were assessed to examine the different dimensions of breathing pattern dysfunction.

MARM measures the thoracic and abdominal movements during breathing [17]. The procedure is explained in supplementary material (Supplementary material 1). It involves 3 components namely the MARM volume, the MARM balance and the percentage ribcage motion. The reliability and internal consistency of the balance and percentage ribcage motion components are high with *r* = 0.85, Cronbach’s α = 0.85 and *r* = 0.84, Cronbach’s α = 0.84, respectively. However the reliability of the volume component is low (*r* = 0.13) [17].

Breath hold test was used to assess biochemical component of breathing dysfunction. The participants were told to sit and relax and perform normal breathing. Then, they were asked to breathe out and then hold (end expiratory hold), till the point of discomfort or till the first sense of difficulty. A stopwatch was used to record the timing of breath hold. Three such maximal breath holds were performed at three minutes rest intervals each. The mean of three readings were used to determine the results [18, 19].

SEBQ was used to determine the breathing related symptoms. It is a self reported outcome with 25 items, each scored from 0 (never/not true at all) to 3 (frequently/very true). A total score greater than 11 indicates breathing problem. It has a high test – retest reliability [intraclass correlation coefficient (3, 1) = 0.89; 95% CI 0.85–0.92], internal consistency (Cronbach’s a = 0.93) and validity [20].

The HSAT was carried out by a sleep technician and the other outcomes MARM, BHT and SEBQ were assessed by a qualified physiotherapist. All the outcome assessors were blinded to the objective of the study.

## Sample size calculation

The sample size was calculated using G*power (version 3.1.9.7). With a two-tailed α = 0.05, power = 0.80, and a medium effect size (ρ = 0.30), the minimum required sample size was 85 participants. Considering that five correlations were planned, the α level was adjusted using Bonferroni correction (0.05/5 = 0.01), increasing the required sample to approximately 110 participants. So, 120 participants were planned to ensure adequate power.

### Statistical analysis

The statistical analysis was performed using jamovi (version 2.3.28). The results were reported as mean ± SD for quantitative variables including age, BMI, AHI, ODI, MARM volume, balance, percentage ribcage motion, BHT and SEBQ scores. Relative percentage and frequency were used for gender. Independent t test was used for descriptive analysis and Shapiro-Wilk test to check for normality. The variables in the study showed non-normal distribution. Correlation matrix with Spearman’s rho was used to analyze the correlation between the variables. The statistical significance was set as *p* < 0.05. Multiple linear regression analysis, adjusting for age, BMI and gender, was performed to determine the independent effect of dysfunctional breathing outcomes on AHI. Additionally, binomial logistic regression was performed to examine the predictors distinguishing moderate and severe OSA after adjusting for age, BMI and gender.

A secondary analysis was performed to compare the means of MARM volume, balance, percentage ribcage motion, BHT and SEBQ scores for moderate OSA and severe OSA groups. Independent t test or its non-parametric variants were used for the analysis. The effect size was reported using Cohen’s d.

## Results

A total of 120 patients with the mean age of 47.5 ± 13.88 were included in the study. The demographic details of the participants are reported in Table 1.

**Table 1:** Demographic details of the participants

Outcomes	Mean ± SD/Frequency (*n* = 120)
Age (years)	47.5 ± 13.88
Gender	Male: 70% (*n* = 84) Female: 30% (*n* = 36)
BMI (kg/m^2^)	29.6 ± 4.94
AHI (events/hour)	34.8 ± 17.22
ODI	42.3 ± 21.83
MARM Volume (degrees)	69.7 ± 4.42
MARM Balance (degrees)	32.8 ± 9.04
MARM Percentage Ribcage Motion (%)	73.5 ± 6.13
Breath Hold Test (seconds)	15.6 ± 4.24
SEBQ Score	32.9 ± 9.55

n, number of participants; SD, Standard deviation; BMI, Body mass index; AHI, Apnea hypopnea index; ODI, Oxygen desaturation index; MARM, Manual assessment of respiratory motion; SEBQ, Self evaluation of breathing questionnaire

**Fig. 1:**
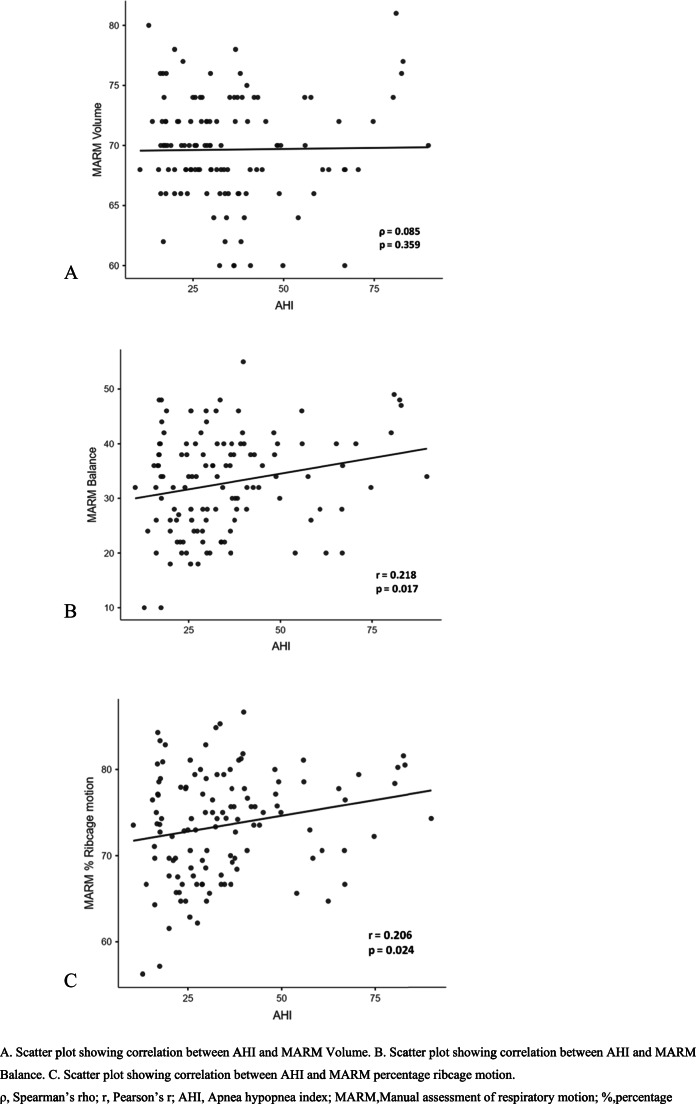
Correlation between apnea hypopnea index and manual assessment of respiratory motion components

**Fig. 2:**
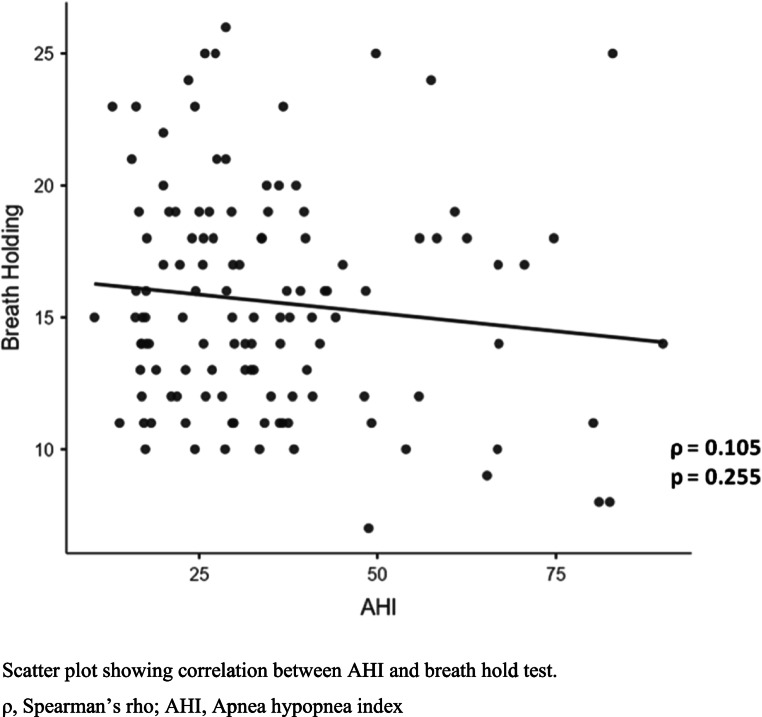
Correlation between apnea hypopnea index and breath hold test

**Fig. 3:**
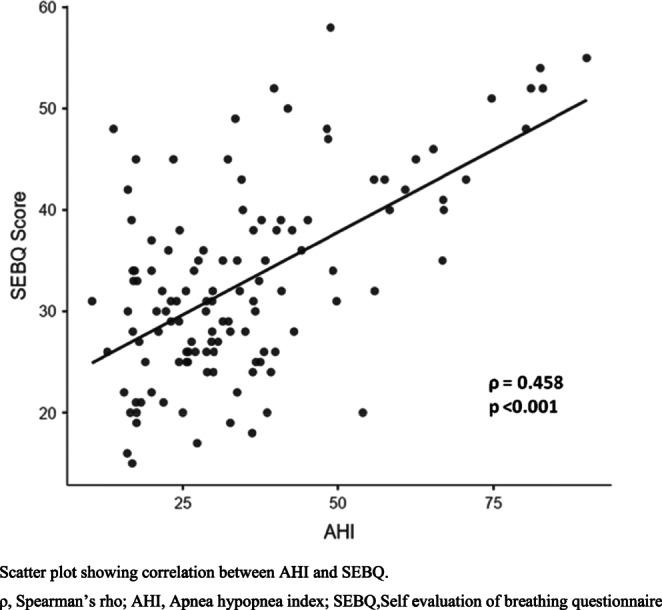
Correlation between apnea hypopnea index and self evaluation of breathing questionnaire

Table 2 shows the results of the correlation of age, BMI and dysfunctional breathing with severity of OSA. Age showed no correlation (ρ = −0.006) and BMI presented with a significant but weak positive correlation (ρ = 0.028) with AHI. The MARM components showed mixed results with negative but non-significant correlation between volume and AHI (ρ = −0.085), however, there was significant correlation of balance and percentage ribcage motion. The balance between ribcage and abdominal motion demonstrated weak positive correlation with AHI (ρ = 0.187) suggesting an increase in the difference between ribcage and abdominal motion with increased severity of OSA. Additionally, the percentage ribcage motion also showed a weak positive correlation with AHI (ρ = 0.205) suggesting an increase in the predominant ribcage motion with increased severity of OSA. The BHT showed non-significant negative correlation with AHI (ρ = −0.105). Furthermore, SEBQ parameter exhibited significant results with moderate positive correlation with AHI (ρ = 0.458). The scatter plots presenting the relation of dysfunctional breathing parameters with severity of OSA are presented in Figs. 1, 2 and 3.

**Table 2:** Correlation of outcomes with apnea hypopnea index (*n* = 120)

Outcome	Correlation coefficient	*p* value
Age	−0.006	0.94
BMI	0.028	0.002*
MARM Volume	−0.085	0.359
MARM Balance	0.187	0.041*
MARM percentage ribcage motion	0.205	0.025*
Breath hold test	−0.105	0.255
SEBQ Score	0.458	< 0.001*

*p<0.05

n, number of participants; BMI, Body mass index; MARM, Manual assessment of respiratory motion; SEBQ, Self evaluation of breathing questionnaire

On examining the predictors of AHI through multivariable linear regression (Table 3), MARM volume and SEBQ showed significant association (*p* < 0.05) with AHI after adjusting for age, BMI and gender. MARM balance and percentage ribcage motion also reached the borderline with p value of 0.05 and 0.06, respectively. The model was statistically significant (F = 16.7, *p* < 0.001) with 55% variability in AHI (R^2^ = 0.55, Adjusted R^2^= 0.51, F = 16.7).

**Table 3:** Multiple linear regression predicting apnea hypopnea index (*n* = 120)

Predictor	ꞵ	SE	95% Confidence Interval		*p* value
			Lower	Upper	
Age	−0.51	0.10	−0.70	−0.31	< 0.001*
BMI	0.38	0.24	−0.09	0.84	0.11
Gender Male – Female	3.70	2.49	−1.24	8.62	0.14
MARM Volume	−1.95	0.91	−3.76	−0.15	0.03*
MARM Balance	3.81	1.94	−0.04	7.67	0.05
MARM percentage ribcage motion	−5.16	2.74	−10.58	0.27	0.06
Breath hold test	−0.16	0.28	−0.71	0.39	0.57
SEBQ Score	1.33	0.14	1.04	1.61	< 0.001*

Note: Model Statistics: R^2^ = 0.55, Adjusted R^2^= 0.51, F = 16.7, *p* < 0.001

*p<0.05

n, number of participants; AHI, Apnea hypopnea index; BMI, Body mass index; MARM, Manual assessment of respiratory motion; SEBQ, Self evaluation of breathing questionnaire; ꞵ,coefficient; SE, Standard error

Further, the results of the logistic regression for severe OSA and moderate OSA revealed that SEBQ is a significant predictor of OSA (OR = 1.17, *p* < 0.001). Also, the age and BMI showed significant association (*p* < 0.05) with AHI (Table 4).

**Table 4:** Binomial logistic regression predicting severe OSA (*n* = 60) vs. moderate OSA (*n* = 60)

Predictor	ꞵ	SE	Odds Ratio (OR)	95% Confidence Interval of OR		*p* value
				Lower	Upper	
Age	−0.06	0.02	0.94	0.90	0.99	0.01*
BMI	0.11	0.05	1.11	1.01	1.00	0.03*
Gender Male – Female	0.22	0.53	1.24	0.44	3.53	0.68
MARM Volume	−0.33	0.28	0.72	0.42	1.25	0.25
MARM Balance	0.30	0.55	1.34	0.45	3.99	0.59
MARM percentage ribcage motion	−0.33	0.77	0.72	0.16	3.22	0.66
Breath hold test	−0.06	0.06	0.94	0.83	1.07	0.36
SEBQ Score	0.16	0.04	1.17	1.08	1.27	< 0.001*

Note: Model Statistics: Deviance = 113, AIC = 131, McFadden’s R^2^= 0.32, AUC = 0.87

*p<0.05

n, number of participants; AHI, Apnea hypopnea index; BMI, Body mass index; MARM, Manual assessment of respiratory motion; SEBQ, Self evaluation of breathing questionnaire; ꞵ,coefficient; SE, Standard error

The secondary analysis (Table 5) demonstrated significant differences in MARM and SEBQ parameters among patients with moderate and severe OSA (*p* < 0.05) with a moderate to large effect size. However, there were no significant differences in BHT results between both the groups (*p* = 0.09) (Supplementary Material 2).

**Table 5:** Comparison of outcomes between moderate and severe OSA groups

Outcomes	Moderate OSA (*n* = 60)		Severe OSA (*n* = 60)		*p* value	Effect Size
	Mean ± SD	Median (Q_1_,Q_3_)	Mean ± SD	Median (Q_1_,Q_3_)		
Age	47.3 ± 13.63	46.0 (35.0, 59.0)	47.6 ± 14.24	46.0 (37.8, 57.5)	0.88	0.02
BMI	28.2 ± 4.54	27.4 (25.2, 30.4)	31.0 ± 4.96	30.4 (27.8, 33.3)	0.001^a^*	0.59
MARM Volume	70.5 ± 3.38	70.0 (68.0, 72.0)	68.8 ± 5.13	68.0 (66.0, 72.5)	0.04^a^*	0.41
MARM Balance	31.0 ± 9.36	32.0 (24.0, 38.0)	34.6 ± 8.40	36.0 (28.0, 40.0)	0.02^b^*	0.41
MARM percentage ribcage motion	72.0 ± 6.59	72.5 (66.7, 77.3)	75.0 ± 5.27	75.3 (70.6, 78.8)	0.006^c^*	0.51
Breath hold test	16.2 ± 4.27	15.5 (13.0, 19.0)	14.9 ± 4.14	15.0 (12.0, 18.0)	0.09^b^	0.30
SEBQ Score	28.9 ± 7.05	28.5 (25.0, 33.0)	36.8 ± 10.15	37.0 (28.8, 43.5)	< 0.001^c^*	0.90

a, Mann-Whitney U; b,Student’s t; c,Welch’s t; *p<0.05

n, number of participants; SD, Standard deviation; MARM, Manual assessment of respiratory motion; SEBQ, Self evaluation of breathing questionnaire

(A) Scatter plot showing correlation between AHI and MARM Volume. (B) Scatter plot showing correlation between AHI and MARM Balance. (C) Scatter plot showing correlation between AHI and MARM percentage ribcage motion

ρ, Spearman’s rho; r, Pearson’s r; AHI, Apnea hypopnea index; MARM, Manual assessment of respiratory motion; %,percentage

Scatter plot showing correlation between AHI and breath hold test

ρ, Spearman’s rho; AHI, Apnea hypopnea index

Scatter plot showing correlation between AHI and SEBQ

ρ, Spearman’s rho; AHI, Apnea hypopnea index; SEBQ, Self evaluation of breathing questionnaire

## Discussion

The primary findings of the study suggest that the biomechanical component and patient reported symptoms of dysfunctional breathing are markedly altered in patients with moderate to severe OSA. The volume of breathing showed minimal negative correlation with severity of OSA. However, the difference between ribcage and abdominal motions and the percentage of ribcage motion denoted notable alterations in breathing among these patients. In addition to this, the patient reported breathing symptoms were substantially increased with the severity of OSA. In contrast to these results, the biochemical component failed to show consequential deteriorations in patients with OSA but demonstrated a slight negative correlation.

The secondary findings suggest noteworthy differences in the dysfunctional breathing outcomes between individuals with moderate and severe OSA. According to the present study, the alterations in biomechanical aspect of breathing are more pronounced in the patients with severe OSA with a considerable decrease in the ribcage and abdominal motions and predominant thoracic breathing as compared to the patients with moderate OSA. However, the breath hold duration failed to report meaningful differences between the patients. Further, the patients with severe OSA reported worse breathing related symptoms as compared to the patients with moderate OSA.

The biomechanical component of dysfunctional breathing in our study suggested that the predominant thoracic breathing was observed among patients with OSA. This can be attributed to several factors among the obese and non-obese population. The decreased end expiratory volume due to obesity and supine positioning with increased diaphragmatic dome causes impaired excursion of diaphragm [21]. This necessitates the use of thoracic muscles leading to heightened reliance on the less efficient thoracic expansion. Further, the respiratory mechanics are distorted by high negative intrathoracic pressures caused by upper-airway obstruction [10]. Particularly when sleep-onset hypotonia reduces overall muscle efficiency, subtle pharyngeal collapsibility (elevated Pcrit) causes early upper-airway constriction during sleep, resulting in large negative intrathoracic pressures that preferentially activate thoracic intercostals over the diaphragm [22]. Asynchronous thoraco-abdominal patterns, in which thoracic excursion predominates are fostered by high loop gain and low arousal threshold [10].

These nocturnal maladaptations cause altered breathing throughout the day [7, 8]. Spirometry or optoelectronic plethysmography for awake patients with OSA frequently shows thoracic dominance with high respiratory rate variability [12, 23]. Despite having a normal BMI, patients report mild dyspnea upon exertion and reduced VO_2max_ due to increased muscle effort as the thoracic muscles are less efficient than diaphragm for tidal volume [24].

Moreover, after adjusting for the potential confounders of age, BMI and gender, the biomechanical component of dysfunctional breathing, MARM volume, contributed to the OSA severity. However, the role of these predictors in differentiation between moderate and severe OSA remains limited.

In the current study, MARM Balance component showed positive correlation with OSA severity. It depicts that the there was increased difference in the ribcage and abdominal motion with increasing severity. The possible explanation could be increase in the predominant ribcage movement and a reduced abdominal movement with increasing severity of OSA.

The biochemical aspect of dysfunctional breathing showed minimal but negative trend with severity of OSA suggesting a decrease in the duration of breath hold with increasing severity among patients with OSA. It also lacked association with AHI after adjusting the potential confounders in the current study. Evidence suggests that the severity of OSA influences chemoreflex sensitivity and loop gain [19]. However, patients with a different phenotype of OSA with overlapping hypoventilation exhibit blunted chemosensitivity [19]. These patients may paradoxically present with a higher duration of breath hold capacity obscuring the biochemical component of dysfunctional breathing. This could be the possible reason behind the non-significant relationship between BHT and AHI. Moreover, BHT is an indirect measure of the chemoreflex sensitivity, therefore, its prediction about dysfunctional breathing could be limited [25].

The current study demonstrated positive relationship between the patient-reported symptoms and severity of OSA. The subjective symptoms of dysfunctional breathing showed meaningful contribution in predicting severity of OSA and distinguishing severe OSA from moderate OSA.

The altered thoraco abdominal mechanics with a predominant thoracic breathing observed among patients in the current study indicate an inefficient reliance on accessory muscles for breathing. The patients may perceive breathlessness during physical activity or laboured breathing and chest tightness [26]. These SEBQ items may lead to a rise in the total score. Moreover, the shift in the chemoreflex sensitivity could manifest as shallow breathing or frequent sighing and yawning among the patients with OSA further increasing the SEBQ scores [27]. Additionally, a majority of patients in our study reported mouth breathing at night and sometimes during day as well. Furthermore, the recurrent arousals and sympathetic surges from OSA could lead to hypervigilance to breathing sensations [28]. This could affect the emotional and cognitive aspects of SEBQ, such as anxiety or worry about breathing.

Our results showed an inverse relationship between age and OSA severity, despite the fact that OSA prevalence is known to rise with age. This could be due to the greater number of younger individuals in the current study. Therefore, this finding should be interpreted with caution.

Further, the comparison of dysfunctional breathing parameters in individuals with moderate and severe OSA revealed worsened biomechanical and patient reported outcomes in patients with severe OSA. The results could be attributed to the increased ventilator instability, heightened chemosensitivity and large irregularities in breathing pattern with marked thoraco-abdominal breathing in patients with severe OSA as compared to the patients with moderate OSA [29].

## Strengths and limitations

The present study is the first one to determine the relationship of all the three aspects of dysfunctional breathing involving biomechanical, biochemical and patient reported symptoms of breathing among patients with OSA. Additionally, the studies till now assessed the breathing behaviors through polysomnography during sleep. However, evaluation in current study occurred in daytime during a stable resting state thereby minimizing the confounding factors such as sensor attachments, pre sleep anxiety during the test, lab environment and position changes could limit the findings. Moreover, the dysfunctional breathing parameters used in the study are cost effective and feasible to perform among patients. According to the recent evidence, these symptoms are not treatable by CPAP alone necessitating a different therapeutic approach for addressing dysfunctional breathing in patients with OSA [30].

There are few limitations in the study. First, the cross sectional nature of the study limits the causal inference. Future longitudinal studies should address the temporal relationship between OSA and dysfunctional breathing. Next, the heterogeneity of OSA population was not adequately addressed through subgroups in our study. The patients with different phenotypes such as positional OSA or REM dependent OSA and patients in different BMI categories, waist and neck circumferences, age and gender may be addressed in future studies

## Conclusion

The study reported alterations in breathing during daytime in patients with OSA. The changes in biomechanical aspect of dysfunctional breathing with a predominant thoracic motion, and patient reported symptoms on the breathing behavior, were markedly related to the severity of OSA. The volume of respiratory movement and subjective symptoms were independently associated with severity of OSA after adjusting for the age, BMI and gender. Additionally, the subjective symptoms showed good discriminative ability for severe OSA. The secondary findings suggested that all the aspects of dysfunctional breathing were substantially affected in individuals with severe OSA as compared to those with moderate OSA.

## Supplementary Information

Below is the link to the electronic supplementary material.

Supplementary Material 1 (DOCX 11.9 KB)

Supplementary Material 2 (DOCX 103 KB)

## Data Availability

The data that support the findings of this study are available from the corresponding author upon reasonable request.

## Declarations

### **Trial registration**

Clinical Trials Registry – India (CTRI/2025/03/082574). 
