# Supplementary material for: Dysfunctional breathing in patients with moderate and severe obstructive sleep apnea: a cross sectional study
Source: Sleep Breath. 2026 May 1;30(2):150. doi: 10.1007/s11325-026-03673-4 (PMC13135013; doi:10.1007/s11325-026-03673-4)
Supplement: Supplementary file 1 — Supplementary Material 1 (DOCX 11.9 KB) [file 11325_2026_3673_MOESM1_ESM.docx]

**Supplementary Material 1**

**Manual Assessment of Respiratory Motion**

For performing the test, the therapist sat behind the subject. The therapist placed both the hands on the lower lateral ribs of the participant and hands spread comfortably with little finger in horizontal orientation and the thumbs remain parallel to the spine pointing vertically. The 4^th^ and 5^th^ fingers reached below the ribs to feel abdominal expansion. The vertical and lateral motion of the hand assessed if the motion is predominantly of upper ribcage, lower ribcage/ abdomen or balanced. The MARM diagram consists of horizontal line and upper and lower lines at relative distance. In case of increased vertical motion of participant’s ribcage, the upper line would be closer to the top and further from horizontal and in case of increased lateral and lower ribcage/ abdomen motion, the lower line would be closer to the bottom and further from horizontal. The MARM measurements included-

1. MARM Volume – It is the angle formed between upper and lower line. The MARM volume measure reflects the overall excursion generated by the combined movements of the ribcage and abdomen. Because it represents the sum of these two contributions, a larger volume score indicates greater total respiratory motion, whereas a smaller value suggests reduced or limited thoraco-abdominal expansion during breathing.

2. MARM Balance – It is the difference between the angles made by horizontal axis and upper line and horizontal axis and lower line. The balance score reflects how different the movements of the ribcage and abdomen are during breathing. When this value increases, it indicates that one region is moving much more than the other, suggesting reduced coordination between thoracic and abdominal components of respiration. A lower score, on the other hand, shows that both areas are expanding in a more comparable and coordinated manner, which is typical of an efficient and well-integrated breathing pattern.

3. Percent ribcage motion – It is the area above horizontal/total area between upper line and lower line * 100. The percentage ribcage motion value expresses how much of the total respiratory movement is contributed specifically by the ribcage. It is calculated as the proportion of ribcage displacement relative to the combined ribcage and abdominal motion. Higher percentages denote a breathing pattern dominated by upper-chest activity, while lower percentages indicate a comparatively greater contribution from the abdomen, which is more typical of efficient diaphragmatic breathing.
