# Supplementary material for: Dysfunctional breathing in patients with moderate and severe obstructive sleep apnea: a cross sectional study
Source: Sleep Breath. 2026 May 1;30(2):150. doi: 10.1007/s11325-026-03673-4 (PMC13135013; doi:10.1007/s11325-026-03673-4)
Supplement: Supplementary file 2 — Supplementary Material 2 (DOCX 103 KB) [file 11325_2026_3673_MOESM2_ESM.docx]

**Supplementary Material 2**

Comparison of means for the dysfunctional breathing outcomes between moderate and severe OSA

1.
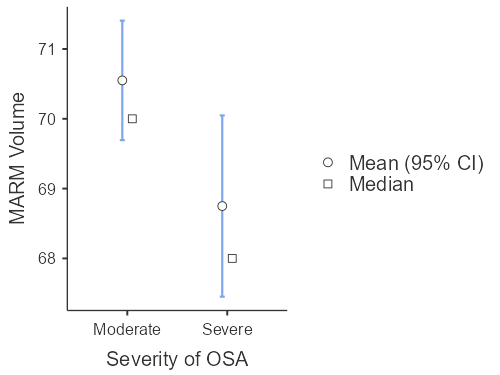

2.
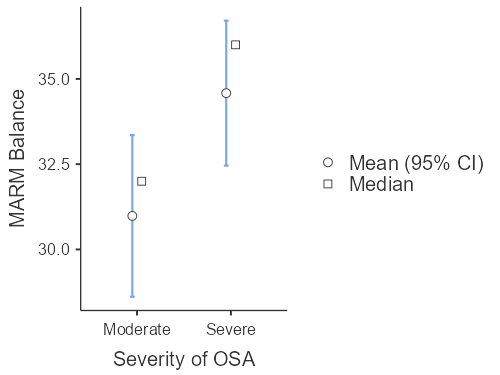

3.
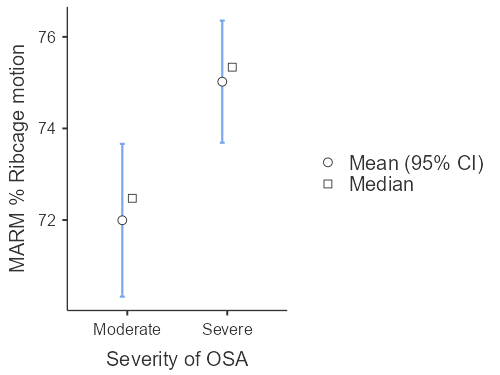

4.
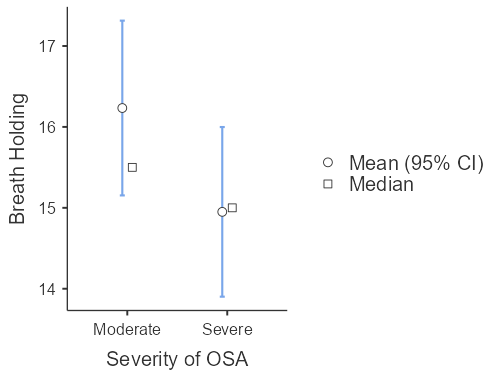

5.
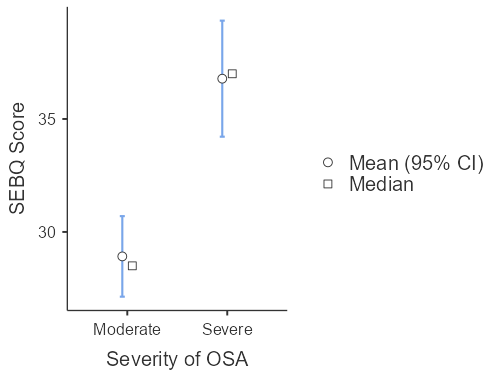


^A. Point estimate plot showing MARM Volume in patients with moderate and severe OSA. B. Point estimate plot showing MARM Balance in patients with moderate and severe OSA. C. Point estimate plot showing MARM percentage ribcage motion in patients with moderate and severe OSA. D. Point estimate plot showing breath holding in patients with moderate and severe OSA. E. Point estimate plot showing SEBQ scores in patients with moderate and severe OSA.^

^OSA,Obstructive sleep apnea; AHI,Apnea hypopnea index; MARM,Manual assessment of respiratory motion; SEBQ,Self evaluation of breathing questionnaire^
